# Adequacy of Head CT Scan Request Form Completion for Non-trauma Neurological Cases at a Teaching Hospital in Baghdad, Iraq: A Clinical Audit

**DOI:** 10.7759/cureus.93088

**Published:** 2025-09-24

**Authors:** Sarmad Al-Hilfi

**Affiliations:** 1 Department of Surgery, College of Medicine, University of Basra, Basra, IRQ

**Keywords:** adequacy of completion of request form, clinical audit, ct scan, neuroimaging, non-trauma neurological cases

## Abstract

Objective

To determine whether the clinical and demographic data recorded on head CT scan request forms for patients with non-trauma neurological issues at the Baghdad Teaching Hospital emergency department are adequate, and to explore if the process of completing radiology request forms can be improved through specific interventions.

Methodology

A clinical audit was conducted in two cycles. Data were collected from head CT request forms for patients presenting with suspected non-traumatic neurological issues to the emergency room. In the first cycle, data were gathered retrospectively over one month in January 2025. The data were reviewed and analyzed, and then interventions were implemented to enhance quality. The second cycle took place over one month (April 2025) to objectively evaluate the success of these interventions by comparing the adequacy of relevant information before and after their implementation.

Results

Four hundred one patients were included in the first cycle, with 65.58% of their requests having inadequate clinical information. After implementing the interventions, 30.28% of the 383 patients in the second cycle had insufficient clinical information. The patient demographics were mainly complete, except for age and the time and date of the CT scan request, which showed significant improvement.

Conclusion

The audit study reveals that the implemented interventions significantly improved the radiology request forms by ensuring they contain adequate demographic and clinical information. Ongoing efforts are essential to sustain good practices in completing the radiology request forms.

## Introduction

CT request forms are an essential means of communication between physicians and radiologists. They are used to request a CT scan, followed by a report from a radiology specialist, which is often utilized to help in diagnosis. It is critical to fill out the request forms with accurate information [1].

Radiologists frequently interpret imaging studies and write reports based on the clinical information provided. Clinical information refers to all details about the patient's health status, such as the current problem, comorbid and past medical history, current medications, allergies, fasting status, suspected diagnosis, and clinical questions to be addressed [2].

The need to provide adequate clinical information on request forms is well recognized and widely discussed in the literature. Multiple meta-analyses have shown that clinical history improves diagnostic accuracy and results in more clinically useful outcomes [3-5]. This is particularly vital in urgent care settings, where providing a concise yet comprehensive clinical history is essential for proper radiology study acquisition and accurate interpretation [5].

Providing clinical information becomes increasingly important when employing advanced imaging, such as CT scans, as the value of clinical information increases with imaging complexity due to the larger volume of images and the expanded list of differential diagnoses. As a result, insufficient clinical information can be a risk factor for missed diagnoses and reduced trust in incidental findings [2]. Additionally, missing demographic data can cause errors in patient identification and delays in delivering reports to the correct destination, which can decrease the value of the report [6].

The main difficulties noted with increased use of advanced imaging include improper application related to patient safety, financial effects, and longer wait times. Although there is no definitive evidence that ionizing radiation from CT causes cancer, reports indicate that CT scans are responsible for 1.5% to 2% of all cancers in the US [7]. Therefore, request forms should provide sufficient clinical information to justify the need for the diagnostic imaging. The general criterion for proceeding with a medical imaging exam is that the expected clinical benefit outweighs the risks involved, including radiation exposure. International radiation protection guidelines mandate that clinical radiologists ensure patient exposure to medical radiation is clinically justified [8].

All medical imaging examinations at Baghdad Teaching Hospital's emergency department (ED) require a referrer to submit a radiology request form. This request form is paper-based and includes the patient's identification information and the type of examination requested. It is also essential that the referrer provide sufficient clinical information to justify the evaluation. The request must be signed and dated by the referrer. This ensures compliance with radiation safety laws and improves workflow efficiency. This good practice is aligned with guidelines from The Royal Australian and New Zealand College of Radiologists (RANZCR) [8-10] and The Royal College of Radiologists (RCR) [6,11,12].

The purpose of this audit was to evaluate and enhance the quality of demographic and clinical information provided in the CT request forms from the ED at Baghdad Teaching Hospital.

## Materials and methods

Study design and data collection

This study was conducted in two cycles on patients presenting with non-traumatic brain issues who were referred for head CT scans from the ED of Baghdad Teaching Hospital during January and April 2025. Data was collected from paper-based CT head scan request forms. The audit team recorded both clinical details and patient demographics on these forms, and the data were reviewed according to best practices for completing radiology request forms, especially in the emergency department. Formal ethical approval is not required for clinical audits under the guidelines of the Ministry of Health in Iraq. However, the radiology department at BTH approved the study to ensure ethical compliance and adherence to institutional protocols. We used IBM SPSS Statistics for Windows, Version 29.0.2.0 (IBM Corp., Armonk, NY), to analyze the data, employing descriptive statistics such as percentages and means. The Chi-square test was used to evaluate the significance of associations, with a p-value of less than 0.05.

Inclusion criteria

Patients presented to the emergency department with non-traumatic brain problems.

Exclusion criteria

The exclusion criteria involved any patient with a head injury.

Methods

The standard for this audit was that the radiology request form should include sufficient clinical and demographic information that identifies the patient and the destination for the report. This aligns with the protocol in the radiology department at Baghdad Teaching Hospital and is recommended worldwide by the Royal College of Radiologists and the Royal Australian and New Zealand College of Radiologists [6,11,12].

The target was 100%: all radiology request forms must include complete clinical and demographic information.

Adequacy of the head CT request form

Each request form was evaluated for the adequacy of both demographic and clinical information by the audit team members. The demographic information included the patient's name, age, and sex. Additionally, the date, time of the head CT request, and the referrer's name and signature were recorded.

Regarding the adequacy of clinical information, the audit team agreed on using a scoring system based on specific criteria. This evaluation considered the clinical background (history and physical exam), the question to be addressed (provisional diagnosis or relevant differentials), and/or investigations (such as relevant blood results). The scoring system assigns three scores: 0, 1, or 2. This scaling system assigns a score of 0 for cases with insufficient or irrelevant clinical information (inadequate clinical information). A score of 2 is given for complete and sufficient information that aids in interpreting the CT head scan by a radiologist (fully adequate clinical information). 1 reflects information that falls between these two mentioned scores (indicates intermediate adequacy). Table 7 in the appendix provides examples of how to apply the scoring system for assessing the adequacy of clinical information.

Audit cycles

In the first cycle, the total sample consisted of 545 CT brain request forms from patients presented to the ER, with only 401 scans meeting our inclusion criteria. This sample size exceeds the minimum number recommended by the Royal College of Radiologists for such studies [6,11].

Following the analysis of cycle 1 data, the audit team, comprising residents from emergency and radiology departments, a radiologist, and an emergency physician, identified key strategies to improve adequacy. They carried out several interventions, starting with the first: sharing audit findings with ED staff during a team meeting. The second intervention involved education by an ED faculty member to all ED faculty and residents, aimed at improving the process of completing radiology request forms and ensuring more accurate interpretations by radiologists. The third intervention was a reminder slide displayed on a large screen in the ED, showing examples of high-quality versus low-quality clinical information, similar to Table 7 of the Appendix. The fourth intervention involved monitoring ED doctors and providing them with feedback on their referring practices.

After implementing these interventions, a second audit cycle (cycle 2) was conducted over 30 days in April 2025 to evaluate their effectiveness. Of 475 reviewed CT scans, 383 met the inclusion criteria.

## Results

Demographic characteristics of the studied patients

During the analysis of patient demographics for both 401 patients in cycle one and 383 patients in cycle two, it was observed that the patients' names and genders, as well as the referrers' names and signatures, are included in all their request forms. The study shows that male patients were slightly more than females, constituting 52.86% in cycle 1 and 54.04% in cycle 2.

The average age of the study population was calculated based on the ages provided. In cycle 1, the mean age was 57.47±17.42 years, with the youngest participant being 15 years old and the oldest 95 years old. In cycle 2, the mean age was 56.57±19.5 years, with an age range of 14 to 92 years. The demographics are shown in Table 1 and Table 2.

**Table 1:** Demographics of patients in cycle 1 The data is presented as n(%).

Cycle 1	Number and percentage	Patient with the mentioned age (number)	Average age (years) (mean±SD)
Total patients	401 (100%)	311	57.47±17.42
Male patients	212 (52.86%)	169	55.91±16.96
Female patients	189 (47.13%)	142	59.32±17.83

**Table 2:** Demographics of patients in cycle 2 The data is presented as n(%).

Cycle 2	Number and percentage	Patient with the mentioned age (number)	Average age (years) (mean±SD)
Total patients	383 (100%)	369	56.57±19.5
Male patients	207 (54.04%)	198	54.87±18.75
Female patients	176 (45.95%)	171	58.54±19.25

Age, date, and time of requesting the head CT scan

Regarding age, the study found that it was not mentioned in 90 head CT scan request forms in cycle one, accounting for 28.93%. In contrast, only 14 patients in cycle two, whose ages were not mentioned, made up 3.65%. This indicates a statistically significant decrease of 25.28% in poor documentation of the patient's age, with a p-value <0.05. The date and time of requesting the CT head scan were documented in only 48 scans in the first cycle, representing 11.97%. In cycle two, this increased to 197 scans, or 51.43%, reflecting a significant rise of 39.46%. Documentation of patient age, date, and time is shown in Table 3.

**Table 3:** Comparison of the demographic information (age, date, and time of head CT scan request) between the two cycles The data is presented as n(%). Statistical analysis was performed using the Chi-square test, with significance set at p<0.05. Chi-square (χ²): 58.48 for documenting age. Chi-square (χ²): 140.19 for documenting the date and time.

Cycle	Total number	Request forms with the mentioned age	Request forms without the mentioned age	P-value	Request forms with the mentioned date and time	Request forms without the mentioned date and time	P-value
Cycle 1	401	311 (77.55%)	90 (22.44%)	<0.0001	48 (11.97%)	353 (88.02%)	<0.0001
Cycle 2	383	369 (96.34%)	14 (3.65%)		197 (51.43%)	186 (48.56%)	

Adequacy of clinical information

Regarding the adequacy of clinical information, Table 4 compares the two cycles based on the scaling system used by the audit team. In the first cycle, 263 request forms scored 0 (inadequate clinical information), accounting for 65.58% of the total study, while 1- and 2-scored request forms are 93 (23.19%) and 45 (11.22%), respectively. In comparison, during cycle 2, requests with inadequate clinical information (score 0) decreased to 116 (30.28%), and requests scoring 1 and 2 increased to 128 (33.43%) and 139 (36.29%), respectively. The p-value is very small (<0.0001), indicating a statistically significant improvement in the adequacy of clinical information following our interventions. The request forms with inadequate clinical information decreased significantly by 35.3%, while those with scores of 1 and 2 increased by 10.24% and 25.07%, respectively. Table 3 illustrates this association.

**Table 4:** Comparison between the two cycles in terms of adequate clinical information The data is presented as n(%). Statistical analysis was performed using the Chi-square test, with significance set at p<0.05. Chi-square value (χ²): 110.23.

Cycle	Total number	Inadequate clinical information	Intermediate adequate clinical information	Fully adequate clinical information	P-value
Cycle 1	401	263 (65.58%)	93 (23.19%)	45 (11.22%)	<0.0001
Cycle 2	383	116 (30.28%)	128 (33.43%)	139 (36.29%)	

Neurological presentation adequacy between the two cycles

Table 5 and Table 6 show the different neurological presentations documented in the request forms of both cycles, focusing on the adequacy of clinical information for each symptom.

**Table 5:** Neurological symptoms documented in the request forms and adequacy of clinical information for each symptom in cycle 1 The data is presented as n.

Neurological presentation	Total number of request forms	Inadequate clinical information	Intermediate adequate clinical information	Fully adequate clinical information
Altered mental state	114	73	33	8
Focal neurological deficit	165	110	29	26
Headache	45	28	15	2
Seizure	37	22	10	5
Syncope	7	3	2	2
Dizziness	5	3	1	1
Vomiting	4	4	0	0
Fatigue	4	3	1	0
Dysphagia	4	3	1	0
Alexia	2	1	1	0
Vertigo	1	1	0	0
Acute placid paralysis	1	0	0	1
No information	12	12	0	0

**Table 6:** Neurological symptoms documented in the request forms and adequacy of clinical information for each symptom in cycle 2 The data is presented as n.

Neurological presentation	Total number of request forms	Inadequate clinical information	Intermediate adequate clinical information	Fully adequate clinical information
Altered mental state	99	27	39	33
Focal neurological deficit	152	52	43	57
Headache	74	23	26	25
Seizure	26	7	12	7
Syncope	9	1	3	5
Dizziness	5	1	1	3
Vomiting	6	1	2	3
Fatigue	0	0	0	0
Dysphagia	3	0	1	2
Ataxia	3	0	1	2
Vertigo	2	0	0	2
Acute flaccid paralysis	0	0	0	0
No information	4	4	0	0

It is shown that the three most common neurological presentations in both cycles were focal neurological deficits, altered mental states (such as loss of consciousness and confusion), and headaches. Regarding adequate clinical information for documenting these presentations, there were significant improvements in cycle 2, with a p-value less than the specified threshold of 0.05.

## Discussion

A clinician and a radiologist can communicate through radiology request forms. The radiologist should be aware of the clinical data, provisional diagnosis, and required examinations to make a radiological diagnosis and justify the need for CT scans, thus avoiding unnecessary scans and radiation exposure. To correctly identify the patient and prevent report mix-ups, the clinician should also include demographic information [6,11,12].

Regarding demographic information, we found that all CT scan request forms in both cycles included the patient's name and gender, along with the referrer's name and signature. However, the patient's age was documented in 311 request forms (77.55%) in cycle 1. This finding is similar to a study conducted in Pakistan [13], which shows that age is documented in 73.33% of the forms. Knowing the age is essential for radiologists to make accurate diagnoses, as certain diseases are more common in specific age groups. Additionally, age helps accurately identify patients, especially in busy EDs and among patients with similar names. The documentation of patient age improved in cycle 2 to 96.34%.

Regarding the date and time of requesting the CT scan, it was documented in only 48 request forms (11.97%). This is a relatively low percentage, which could affect the management plan, as timing is critical for certain conditions (e.g., it is essential to complete the CT scan report for stroke presentation within 1 hour of the request) [14,15]. In cycle 2, we observed a significant improvement in documenting the time and date of requesting the CT scan, reaching 51.43% of request forms.

Regarding the adequacy of clinical information in cycle 1 of the audit, it was observed that 263 (65.58%) of the head CT scan request forms lacked sufficient clinical details that justified the radiation exposure and assisted the radiologist. This could be due to several factors, including the increased availability of CT and defensive medical practices driven by the threat of medical liability (especially among ED physicians), which results in a lower threshold for its use. Additionally, unawareness among ED doctors about the importance of providing adequate clinical information for radiologists and for justifying advanced imaging might be a contributing factor [16].

Our findings show that request forms with sufficient clinical information make up 34.42% (both 1 and 2 scores), which is lower than the rates seen in two studies in Pakistan [13,17], where the adequacy of clinical information was 50.66% and 79.5%, respectively. A study in Nepal [18] found that 77% of the 239 CT requisition forms collected and audited included the date of examination. The examination category was present in 15% of cases. Likewise, clinical diagnosis was included in 64% of cases, tentative diagnosis in 43%, and clinical history in 30%. CT request forms were often filled out inadequately by recommending physicians in Nepalese hospitals. Insufficient information on radiologic requisition forms harms patient care and safety. Raising awareness about the flow of information between the prescriber and the radiology department is crucial to ensure good communication and promote patient safety and access to radiology services.

After implementing our interventions, although we did not reach our goal of 100% completion of the radiology request forms, we made significant progress in documenting clinical information, which indicates that our strategies have been effective. We notably reduced the number of CT scans without adequate clinical information to 30.28%. Simultaneously, requests with sufficient clinical details increased to 69.71%. This improvement was especially evident in the three main neurological presentations: focal neurological deficit, altered mental state, and headache.

These changes align with good practices at the radiology department in Baghdad Teaching Hospital and adhere to recommendations by the Royal College of Radiology. It is in the best interest of radiologists to emphasize the importance of clinical information for reporting by establishing criteria standards to guide the requesting practices of medical imaging referrers [2]. Furthermore, ongoing efforts from the ED are necessary to sustain and enhance these interventions and to implement new strategies. Regular clinical audits are recommended to address this issue.

Limitations

The scoring system, which assigns three scores for the adequacy of clinical information, could be debated since it relies on criteria set by the audit team. However, the audit team reviewed similar studies in the literature and aimed to categorize the clinical information to justify the CT scan exposure and help the radiologist make accurate diagnoses. This scoring system is more comprehensive than what was used in a similar study of clinical information on head CT requisitions from the ED [19].

The study acknowledges that some request forms with poor documentation, categorized as having inadequate clinical information, may have been shared verbally with the radiologist. This indicates that even verbally communicated information should be documented for records and, in case, a second opinion is needed by another radiologist. Another limitation is that we are unsure if this improvement is just a temporary fix. Therefore, it is vital that we continuously review and evaluate our practices to ensure ongoing progress.

## Conclusions

In conclusion, our interventions have improved the quality of demographic and clinical information on head CT scan request forms. Although the clinical information has significantly increased in completeness, there is still room for improvement. To better ensure proper form completion, it is important to address the root cause of the issue. Providing training can help doctors understand the requirements for radiological request forms and their importance in supporting radiologists, which may lead to higher standards. Regular clinical audits are also necessary to address the problem.

## Appendices

**Table 7:** Scoring system for adequacy of clinical information set by the audit team, with examples for ER doctors ER, emergency room

Examples	Adequacy of clinical information in the head CT request form with the scaling system		
Example number	Inadequate clinical information (score=0)	Intermediate adequate clinical information (score=1)	Fully adequate clinical information (score=2)
1	Headache	Headache of 2 hours duration	Severe headache of a few hours' duration with documented other symptoms and past history. Clinical exam: normal BP and temperature, and neurological exam. Provisional diagnosis: subarachnoid hemorrhage
2	Weakness	Right-sided limb weakness. Past history: hypertension	Right-sided limb weakness of 1-hour duration. Past history: hypertension and diabetes mellitus. Clinical exam: BP 180/100. Blood tests: normal random blood sugar. Provisional diagnosis: suspected stroke
3	Altered mental state	Altered mental state of a few hours’ duration, and past history of diabetes mellitus	Altered mental state of a few hours' duration, and past history of hypertension and diabetes mellitus. Exam: GCS=9. Blood pressure=200/130. Lab tests: normal random blood sugar, normal results for other systems (no chest infection or UTI). Provisional diagnosis: suspected intracranial hemorrhage
